# Immunometabolic Activation of Invariant Natural Killer T Cells

**DOI:** 10.3389/fimmu.2018.01192

**Published:** 2018-05-28

**Authors:** Francesca A. Ververs, Eric Kalkhoven, Belinda van’t Land, Marianne Boes, Henk S. Schipper

**Affiliations:** ^1^Laboratory for Translational Immunology, University Medical Center Utrecht, Utrecht, Netherlands; ^2^Department of Molecular Cancer Research, Center for Molecular Medicine, University Medical Center Utrecht, University Utrecht, Utrecht, Netherlands; ^3^Department of Immunology, Nutricia Research, Utrecht, Netherlands; ^4^Department of Pediatric Immunology, Wilhelmina Children’s Hospital, University Medical Center Utrecht, Utrecht, Netherlands; ^5^Department of Pediatric Cardiology, Wilhelmina Children’s Hospital, University Medical Center Utrecht, Utrecht, Netherlands

**Keywords:** immunometabolism, NKT, obesity, atherosclerosis, sphingolipid, AMPK, mTOR

## Abstract

Invariant natural killer T (iNKT) cells are lipid-reactive T cells with profound immunomodulatory potential. They are unique in their restriction to lipid antigens presented in CD1d molecules, which underlies their role in lipid-driven disorders such as obesity and atherosclerosis. In this review, we discuss the contribution of iNKT cell activation to immunometabolic disease, metabolic programming of lipid antigen presentation, and immunometabolic activation of iNKT cells. First, we outline the role of iNKT cells in immunometabolic disease. Second, we discuss the effects of cellular metabolism on lipid antigen processing and presentation to iNKT cells. The synthesis and processing of glycolipids and other potential endogenous lipid antigens depends on metabolic demand and may steer iNKT cells toward adopting a Th1 or Th2 signature. Third, external signals such as toll-like receptor ligands, adipokines, and cytokines modulate antigen presentation and subsequent iNKT cell responses. Finally, we will discuss the relevance of metabolic programming of iNKT cells in human disease, focusing on their role in disorders such as obesity and atherosclerosis. The critical response to metabolic changes places iNKT cells at the helm of immunometabolic disease.

## Invariant Natural Killer T (iNKT) Cells Center Stage in Immunometabolic Disease

Immunometabolic diseases such as obesity, type 2 diabetes, and cardiovascular disease (CVD) are the major health burdens of our time and illustrate the intricate web between metabolic dysregulation and inflammation (1). The links between metabolism and inflammation may be explained from an evolutionary perspective. An effective immune defense critically depends on efficient energy storage and release, as reflected by the co-evolution of the immune system and metabolism in *Drosophila* fat bodies, and the reminiscent immune cell functions of adipocytes in humans and other higher organisms (2). Unfortunately, evolution could not foresee the endemic nutritional overload in 21st century Western societies, causing glucotoxicity and lipotoxicity, and propagating local and systemic inflammation (3).

NKT cells were identified as important players in immunometabolism due to their unique response to lipid antigens and hybrid qualities of both the innate and adaptive immune system (4). NKT cells readily produce copious amounts of Th1, Th2, and/or Th17 cytokines upon activation, which resembles an innate activation scheme (5). Similar to T cells, NKT cells develop in the thymus and undergo positive and negative thymic selection. However, instead of interacting with MHC class 2 molecules, iNKT cells are selected by CD1d-expressing thymocytes. Two NKT cell subtypes have been defined: type 1 signifies CD1d-restricted iNKT cells carrying an invariant T cell receptor that recognizes the prototypic ligand alpha-galactosylceramide, while type 2 signifies CD1d-restricted iNKT cells carrying different T cell receptors not recognizing alpha-galactosylceramide (6). This review focuses on type 1 NKT cells, also known as iNKT cells, which represent the most studied NKT cell subset.

Invariant natural killer T cell frequency in peripheral blood is low, but they are highly enriched in adipose tissue (AT) in mice and humans (7, 8). Functionally, AT-resident iNKT cells have an anti-inflammatory phenotype by secreting IL-4, which contributes to prevention of insulin resistance and AT inflammation (7, 9). In obesity, the protective IL-4 production by iNKT cells is lost, and total iNKT cell numbers in AT and peripheral blood decrease, making leeway for adipose tissue inflammation, insulin resistance, and type 2 diabetes to develop (7–10). The same phenomenon is observed in other metabolic disorders. When comparing human identical twins, of which only one sibling developed type 1 diabetes, diabetic siblings show lower frequencies of iNKT cells. When multiple iNKT clones were compared from the twins, all clones isolated from diabetic siblings produced only IFN-γ upon stimulation, while all clones isolated from the healthy twin produced both IL-4 and IFN-γ (11). In atherosclerosis, a similar decrease in iNKT cell numbers and production of IL-4 is observed in established CVD (12). Notably, iNKT cell numbers in peripheral blood seem to increase in the earliest phase of atherosclerosis, accompanied by an increase in IL-4 production, GATA3- and CD69 expression, and increased proliferative capacity (13). This model, in which iNKT cells play an anti-inflammatory or pro-homeostatic role early in disease development, seems widely applicable for human disease (14), and begs the question: what do iNKT cells see when trouble starts stirring?

## iNKT Cell Activation by Sphingolipid Ligands

In the early 1990s, it was discovered that iNKT cells can be activated by glycosphingolipids (GSL) following identification of alpha-galactosylceramide, a potent marine sponge sphingolipid antigen identified in a cancer antigen screen (15). Since then, endogenous sphingolipids have been scrutinized as potential lipid antigens for iNKT cells.

**Figure 1:**
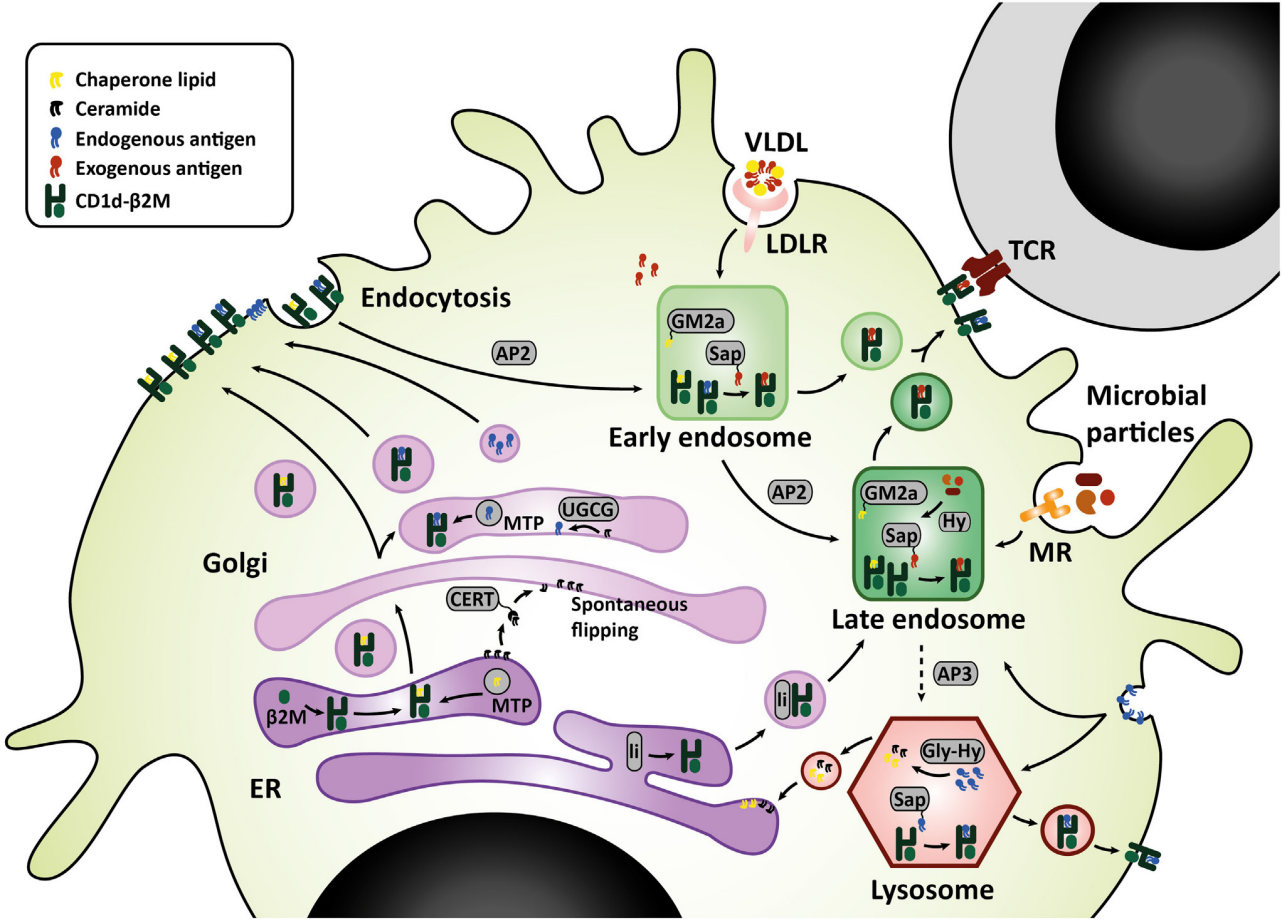
CD1d lipid loading at the crossroads of glycosphingolipid metabolism. In the ER–Golgi pathway that is similar for mouse and human, CD1d heavy chains assemble with β2M and chaperone lipids in the ER before transit to the cell surface. Alternatively, the Golgi complex produces GSL that are loaded onto CD1d by microsomal transfer protein, and may serve as endogenous lipid antigens. Ceramide precursors are transported to the Golgi by ceramide transfer protein (CERT). In the Golgi, UGCG and other glycosyltransferases convert ceramide into GSL. These GSL endproducts can be loaded onto CD1d, or transported to the plasma membrane in membrane-bound transport carriers. In the endolysosomal pathway, mouse CD1d1 may target the endosome and lysosome directly *via* interaction with sorting motifs adaptor protein 2 (AP2), which targets the endosomal compartment, and adaptor protein 3 (AP3), which targets the lysosomal compartment. Human CD1d is internalized *via* AP2 but cannot bind AP3. However, human CD1d can still be found in the lysosomal compartment. Possibly, ER-resident CD1d proteins gain access to the endolysosomal compartment *via* an auxiliary pathway, in conjunction with MHC class II-associated invariant chain (li). In the endolysosomal compartments, CD1d proteins are loaded with exogenous or endogenous lipid antigens, orchestrated by a variety of lipid transfer proteins including GM2 activator (GM2a), saposins A-D (Sap), and Niemann-Pick type C2 protein. Exogenous lipid antigens are delivered to the endosomal compartment *via* endocytosis of LDLR-associated glycolipids, MR-associated microbial lipids, and other scavenger receptors. Some exogenous lipids require processing into antigenic lipids before CD1d-loading, for example, through lipid hydrolases (Hy). Endogenous lipid antigens are delivered to the endolysosomal compartment *via* endocytosis of membrane-associated GSL, which can be loaded onto CD1d or degraded in lysosomes by glycohydrolases (Gly-Hy) and accessory proteins, before recycling to the ER (salvage pathway). Upon lipid antigen loading in the endolysosomal compartments, CD1d–lipid complexes recycle back to the cell surface for interaction with the invariant TCR on invariant natural killer T cells. Abbreviations: AP, adaptor protein; β2M, β2-microglobulin; ER, endoplasmic reticulum; Gly-Hy, glycohydrolase; GM2a, GM2 activator; GSL, glycosphingolipids; Hy, hydrolase; LDLR, LDL-receptor; Li, MHC class II-associated invariant chain; MR, mannose receptor; Sap, saposins; TCR, T-cell receptor; UGCG, UDP-glycose ceramide glucosyltransferase; VLDL, very-low-density lipoprotein.

Sphingolipids are synthesized either *via* the *salvage pathway*, by degradation and re-usage of existing sphingolipids, or *via de novo* synthesis in the endoplasmic reticulum (ER), by attachment of a fatty acid to a sphingosine base (16). Spingomyelinases and glucosidases are important enzymes in the *salvage pathway*, converting membrane sphingomyelin and glucosylceramides back to ceramide within the lysosome (17). Serine palmitoyl transferase (SPT) and ceramide synthases are important for *de novo* synthesis. *De novo* synthesis is orchestrated by six different ceramide synthases (CerS), which determine the length of the fatty acid chain attached to the sphingosine base. Sphingosine with one fatty acid attached is called ceramide, which is the central metabolite in sphingolipid metabolism. More complex sphingolipids such as GSL are generated in the Golgi by addition of different headgroups by UDP-glucose ceramide glucosyltransferase (UGCG) and other glycosyltransferases (18). Translocation to the Golgi is facilitated by ceramide transfer proteins (CERT) (17). The simplest glycosphingolipid has only one sugar residue attached, either glucose or galactose. The sugar headgroup can be attached to ceramide in a beta- or alpha-anomeric fashion. To date, only beta-anomeric GSL have been identified in humans. Some studies reported iNKT cell reactivity to beta-linked GSL, but this was disputed later as contamination of alpha-linkages was found in the preparations (19–22). The alpha-anomeric linkage remains one of the key determinants for antigenicity (20, 23, 24). Enter the search for endogenous lipid ligands continues as, unfortunately, developing a robust method for isolation of these ligands is technically challenging. In the meantime, extensive studies on the effect of various synthetic alpha-galcer analogs on iNKT function were performed, including analogs with truncated alkyl chains, varying saturation status, or the presence of aromatic structures (24–26). These efforts revealed that analogs with a shorter alkyl chain can elicit an IL-4 response without prior IFN-γ induction in mice *in vivo* (alpha-GalCer C10:0, alpha-GalCer C20:2, alpha-GalCer C20:4, OCH, alpha-GalCer-PGB1) (26). However, in human iNKT cells, even though almost all glycolipid analogs elicit a potent cytokine response, there is hardly any Th2-polarization (24, 26). Enter differences between mouse and human ligand-mediated activation are abound: there are differences in potential endogenous ligands and where the ligands derive from, considering that human CD1d and mouse CD1d1 travel to different subcellular compartments for endogenous ligand extraction (27–32). The secretory route from the ER, *via* the Golgi, to the plasma membrane is similar for human CD1d and mouse CD1d1. Upon folding in the ER and association with beta-2-microglobulin, lipid transfer proteins such as microsomal transfer protein mediate loading of chaperone lipids in the ER and/or endogenous lipid antigens in the Golgi (33–35). The endolysosomal recycling route, however, is different for human CD1d and mouse CD1d1. On the basal side of the membrane, CD1d has a short cytoplasmic tail carrying a sorting motif. The sorting motif binds to the adaptor protein complex 2 upon which membrane internalization is mediated to enter the early endosome (36). Only mouse CD1d1, but not human CD1d, can also bind adaptor protein 3, which then targets late endosomes and lysosomes (31). Considering the observed co-localization of human CD1d with the lysosomal membrane protein LAMP1, the lack of a lysosomal sorting motif does not preclude lysosomal transportation of human CD1d (31, 32). Nevertheless, differences in endolysosomal trafficking may result in loading of different lipid antigens. LDL receptor (LDLR)-mediated uptake of GSL for example, is processed in the endosomal compartment (37), while the *salvage pathway* of plasma membrane GSL starts in the lysosomal compartment (38) (Figure 1). These differences are important to keep in mind when studying iNKT cells and Th1/Th2 skewing in mouse models.

## Sphingolipids in Immunometabolic Disease

Sphingolipids play a key role in immunometabolic disease, which supports their potential relevance as iNKT cell antigens (17, 18, 39). The six CerS involved in *de novo* sphingolipid synthesis are differentially expressed, allowing for tissue- and cell type-dependent variation in ceramide acyl chain length profiles (16). Importantly, differences in sphingolipid chain length may orchestrate glucose metabolism and mitochondrial homeostasis, and play a key role in obesity and type 2 diabetes. For example, reduction in C16 sphingolipid levels increases beta-oxidation and improves glucose metabolism, with a 30–50% reduction in C16 levels being sufficient to prevent diet-induced obesity and insulin resistance (40). Furthermore, acyl chain length may determine cell fate. While C22-24 ceramides prevent apoptosis, C16 ceramides can induce apoptosis *via* activation of the intrinsic mitochondrial apoptotic pathway (16, 41–43). In CVD, C16 ceramides are considered harmful, as Cer(d18:1/16:0)/Cer(d18:1/24:0) ratios predict cardiovascular death (44). Intriguingly, sphingolipids such as glucosylceramide, lactosylceramide, ceramide, dihydroceramide, sphingomyelin, and sphingosine-1-phosphate (S1P) amass in human atherosclerotic plaques. All except S1P induce apoptosis *in vitro*, and are associated with plaque instability (39, 45, 46). Consequently, D-PDMP, an inhibitor of glucosylceramide synthase and lactosylceramide synthase, has an astounding protective effect on atherosclerosis development in ApoE^−/−^ mice. Treatment led to complete prevention of intima media thickening and arterial stiffening measured as aortic pulse-wave velocity (47). Likewise, treatment with the SPT inhibitor myriocin was shown to ameliorate insulin resistance and atherosclerosis in mouse and rat models (18).

It is tempting to speculate that the pathophysiological role of sphingolipids in immunometabolic disease is partly explained by their role as iNKT cell ligands. In order to identify sphingolipid antigens potentially involved in immunometabolic disease, several approaches may be explored. First, the intracellular crossing between the sphingolipid metabolism and the iNKT cell lipid loading pathways can be scrutinized (48, 49). The Golgi and lysosomal compartment facilitate exchange of chaperone lipids bound to CD1d for antigenic lipids and are, therefore, important crossroads in iNKT cell lipid antigen loading and sphingolipid metabolism (Figure 1). Alternatively, animal or cellular models of naturally occurring disorders in sphingolipid metabolism may be exploited to identify metabolic intermediates or end products in lipid antigen presentation. For example, the mouse model for Fabry disease, alpha-galactosidase A knock out, combined with globoside 3 synthase- or isogloboside 3 synthase double knock out, revealed that globosides, but not isoglobosides, are responsible for iNKT cell deficiency in Fabry disease (50). Hexb knock out mice, a model for Tay Sach and Sandoff disease, also show severe iNKT cell deficiency (51). The iNKT cell deficiency in these lysosomal storage disease mouse models suggests that the glycosphingolipid synthetic pathways involved may contain endogenous lipid antigens for iNKT cells. Alternatively, glycosphingolipid accumulation may hinder antigen presentation similarly to acLDL accumulation or cholesterol accumulation following NPC1 deficiency (50), and possibly NPC2 deficiency (52), regardless of the glycosphingolipid involved (50, 53). The latter model aligns with the lipid raft hypothesis, which proposes that iNKT cell activating lipids may either function as *bona fide* lipid antigens, *or* may impact CD1d loading, stabilization or clustering on the cell membrane, and in that way enforce iNKT cell activation (50, 54–56). Finally, immunometabolic diseases may serve as a starting point to identify sphingolipid antigens (18). In CVD, for example, lipoprotein particles that enter the cell *via* LDL receptor- and scavenger receptor-mediated uptake are important carriers of glycosphingolipid species (39). The increased uptake of oxidized lipoproteins *via* class A scavenger receptors in atherosclerosis may potentially induce a different iNKT cell effector response due to co-transported glycosphingolipid species (37). In conclusion, sphingolipids are promising candidate antigens from an immunometabolic perspective. However, translation of the changes in sphingolipid metabolism to iNKT cell activation remains technically challenging.

## Indirect Activation of iNKT Cells

At present, two principal ways of iNKT cell activation have been described. As discussed before, high affinity lipid antigens may induce a strong T-cell receptor (TCR) signal and activate iNKT cells directly. Alternatively, innate activation of an antigen-presenting cell (APC) leads to presentation of an endogenous lipid ligand with low affinity, followed by a weak TCR signal that can fully activate iNKT cells in combination with cytokine co-stimulation secreted by the activated APC (5, 57–60). Innate activation of the APC can either be due to inflammatory or metabolic cues. For example, LPS can trigger iNKT-cell activation. This activation is CD1d- and APC dependent. However, this activation is also IL-12 dependent, in both mice and in human *in vitro* models (57, 58). The current model is that iNKT cells are first triggered *via* their TCR to upregulate CD40L, to enhance APC–iNKT cell interaction, and maintain proximity for paracrine IL-12 co-stimulation, which is induced by CD40:CD40L interaction (23, 61, 62). It was postulated that the duration of TCR triggering determines CD40L upregulation. Duration of TCR triggering, in turn, depends on the alkyl chain length and stabilization of the CD1d–glycosphingolipid-complex (24, 25). Furthermore, IL-12 ultimately drives a Th1-biased iNKT cell response (62). *In vivo*, IL-4 can be detected 2 h after intra-peritoneal lipid agonist injection, while IFN-γ is measured after 6 h, as is IL-12 (61). The relatively slow IFN-γ response, which also requires prolonged and enhanced APC–iNKT cell interaction, suggests that the IFN-γ response requires *de novo* IFN-γ protein synthesis, while IL-4 is pre-synthesized and can, therefore, be released instantly even upon weak or short TCR stimulation. In fact, binding affinity of glycolipids to CD1d correlates very well with IFN-γ production but not at all with IL-4 production by human iNKT cells (63). Transgenic mouse studies revealed that the Notch and RBP-J pathway might be responsible for the IL-4 response by iNKT cells, mainly regulated by the conserved noncoding sequence-2 enhancer (CNS-2). As Notch- and TCR signaling synergistically contribute to T cell activation, this could explain why a weak TCR signal still allows for a relatively high IL-4 production by iNKT cells (64). This leaves us with a model in which iNKT cells are potent effector memory IL-4 producers upon homeostatic, weak antigenic stimulation, which can become highly inflammatory IFN-γ producing cells in an environment in which either high affinity ligands are available, or where IL-12 or CD40:CD40L co-stimulation are more easily established, either directly or due to activation of APCs.

## Immunometabolic iNKT Cell Activation

**Figure 2:**
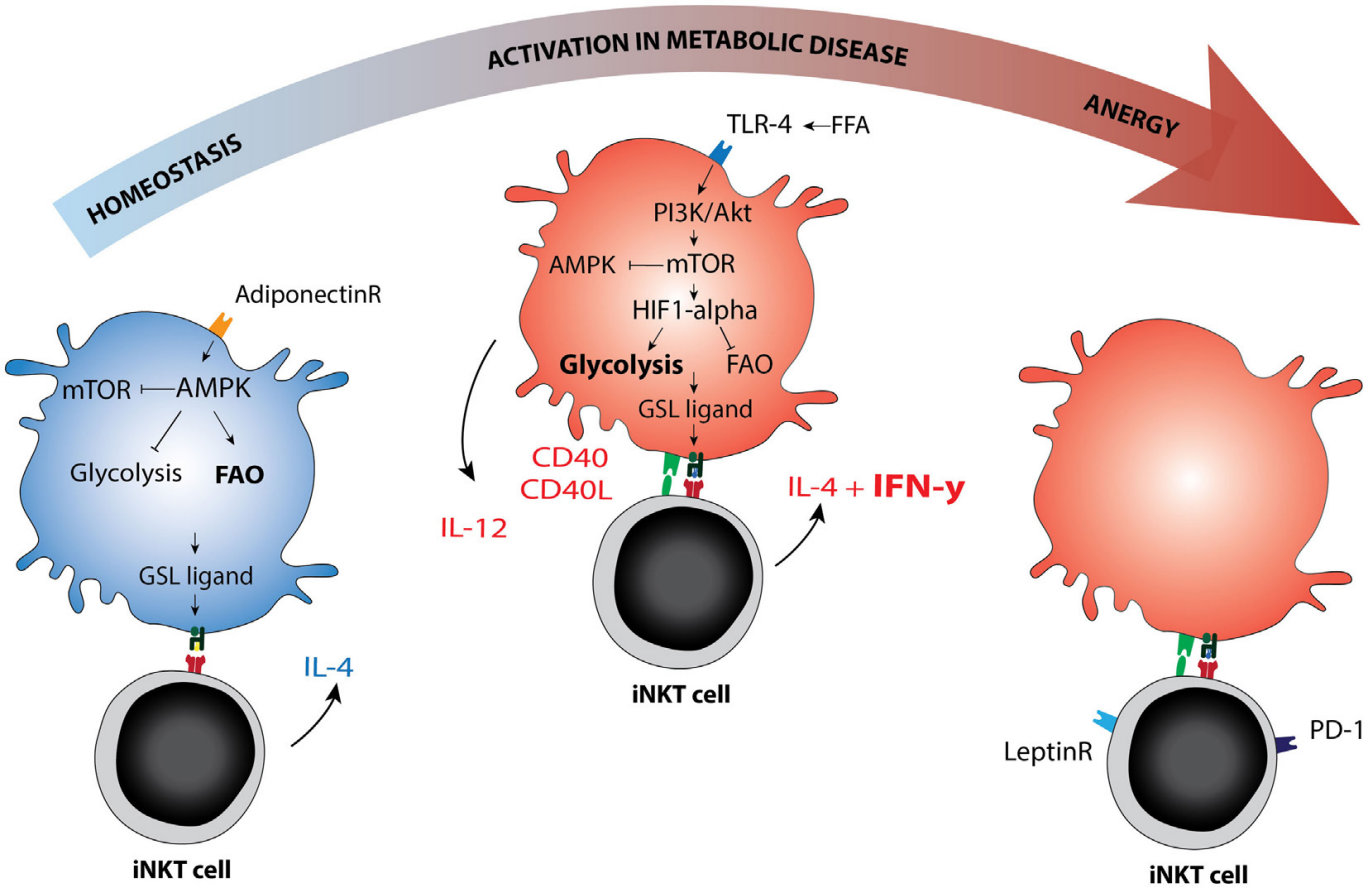
Immunometabolic activation of invariant natural killer T (iNKT) cells. Schematic representation of metabolic re-programming of iNKT cell function in immunometabolic disease. Metabolic changes in the antigen-presenting cell may affect the GSL ligand pool or alter the availability of co-stimulatory molecules. AMPK versus mTOR are depicted as the main metabolic regulators of the cellular metabolic program, they can each inhibit the other. AMPK is activated in low glucose conditions and can be activated by adiponectin, the adipokine secreted by lean adipocytes. AMPK activation drives fatty acid oxidation and is associated with cellular longevity. In lean fat- or homeostatic conditions, the iNKT cell response is mainly Th2 skewed (IL-4), but changes to Th1 in metabolic disease (IFN-γ). Whether AMPK or mTOR activation can be linked directly to Th1 or Th2 skewing of the iNKT cell response has not yet been studied to our knowledge. In dyslipidemic conditions, the hallmark of metabolic disease, FFA may activate mTOR *via* TLR4 signaling. The TLR4-driven glycolysis observed in metabolic disease is reminiscent of glycolysis observed under normoxic conditions in cancer (the Warburg effect). mTOR blocks AMPK and in that manner removes at least one of the brakes on CD40 upregulation and IL-12 production, the co-stimulatory requirements for a Th1 iNKT cell response. In late stage metabolic disease, iNKT cells have been described as anergic, while simultaneously upregulating PD-1. Blocking the leptin receptor on iNKT cells may reverse this anergic state. Abbreviations: GSL, glycosphingolipid; FFA, free fatty acids.

The intracellular sphingolipid pool and subsequent CD1d ligand loading may be affected by TLR-activation or altered metabolism in the APC (65, 66). Several mechanisms were recently reported. For example, blocking glycolysis and increasing fatty acid oxidation (FAO) *via* AMPK provokes a CD1d-mediated iNKT cell cytokine response (65). AMPK is a nutrient-sensing kinase that is activated under low glucose conditions and blocks cellular glycolysis while promoting cell-sparing oxidative phosphorylation (67). Adiponectin, an adipokine produced by lean adipocytes that promotes insulin sensitivity, can directly activate AMPK (65, 68). Adiponectin overexpression in *ob/ob* obese mice protects against insulin resistance and AT inflammation (69), perhaps activating iNKT cells in a Th2-skewed manner through direct or indirect iNKT cell modulation. Conversely, TLR signaling leads to increased glycolysis, reduced FAO, and AMPK inhibition in the APC (70), but again leads to iNKT cell activation (58, 71). TLR-induced glycolysis is established *via* HIF-1α upregulation despite normoxic conditions, analogous to the Warburg effect (70). This pathway may be potentiated by mild hypoxia (72). In early obesity, relative hypoxia arises following adipocyte hypertrophy and hyperplasia and has been dubbed one of the initiating events in AT inflammation (1, 73). Indeed, hypoxia is also an important factor in cancer and in atherosclerosis (72, 74). iNKT cells are sensitive to HIF-1α activation and respond with a CD1d-mediated cytokine response (65). If and how the iNKT cell response is skewed toward an anti- or pro-inflammatory phenotype in these experiments, and whether different ligands are presented, remains to be determined. Notably, TLR4 signaling enhances atherosclerosis development in ApoE^−/−^ mice in an iNKT cell-dependent manner (75). TLR4 signaling can be activated by LPS but also by excess free fatty acids, suggesting that nutrient overload mimics infection with regard to its downstream effects. Furthermore, during obesity, adipocytes produce the adipokine leptin to flag nutrient excess and diminish food intake. Leptin contributes to an iNKT cell response that results in anergy and PD-1 upregulation by directly triggering the leptin receptor expressed by iNKT cells (76, 77). Importantly, leptin-mediated iNKT cell activation still requires TCR triggering (77). These findings support the view that changing metabolic conditions determine the ligand pool and steer the iNKT cell response (Figure 2).

Besides the sphingolipid ligand pool, metabolic changes may also affect co-stimulatory molecules involved in iNKT activation, including CD40 and CD40L. For example, the amount of surface-expressed CD40 on macrophages and smooth muscle cells in human plaques correlates with the stage of atherosclerosis development (78). Possibly, ox-LDL signaling *via* LOX-1, a receptor for ox-LDL, is responsible for the CD40/CD40L upregulation (79). In addition to establishing a firm APC–iNKT cell interaction for Th1 skewing, CD40:CD40L signaling induces LDLR upregulation in human B-cells, enhancing iNKT cell activation (80). Low glucose conditions and AMPK activation causes lower baseline expression of CD40 by dendritic cells, decreased CD40 upregulation, and decreased IL-12 production upon LPS challenge (81). Moreover, AMPK inhibition leads to enhanced IL-12 production after LPS stimulation (81). IL-12 contributes to the formation of early atherosclerotic lesions in ApoE^−/−^ mice and correlates positively with pulse wave velocity in healthy individuals, supporting a role for IL-12 in early atherogenesis in humans (82, 83).

## Implications for Immunometabolic Disease

Immunometabolic diseases such as obesity, type 2 diabetes, and CVD are increasingly considered to be the downside of co-evolution of the immune system and metabolism (2). The growing body of immunometabolic diseases and the intricate web between metabolic dysregulation and inflammation emphasizes the need to understand metabolic programming of immune cells.

Current treatment for immunometabolic disease often targets dyslipidemia, with statins as mainstream therapy. However, we are learning now that the definition of dyslipidemia should extend far beyond cholesterol and triglyceride ratios, since ceramide and sphingolipid metabolism are closely involved in dyslipidemia and its consequences (17, 18, 39, 46). We are just starting to adequately link sphingolipid metabolism directly to metabolic disease and iNKT cell function, but the findings highlighted in this review indicate that the sphingolipid–iNKT cell axis holds promise for new treatment strategies. iNKT cells are unique in their restriction to lipid antigens and seem to possess all qualities required for immune-modulation. However, it is essential to unravel the underlying mechanism(s) directing the iNKT cell cytokine response, and to finally identify the endogenous lipid ligands involved. To this end, we may further explore the microbiome for extraction of potential lipid antigens, as iNKT cell–microbiome interaction has been firmly established (84–87). Additionally, we may further investigate naturally occurring iNKT cell subsets that skew toward Th1 or Th2 cytokine production, Th1/Th2 tissue distribution, plasticity, and the role of epigenetic memory (88, 89). Finally, co-stimulatory molecules, cytokines and adipokines involved in iNKT cell activation may be equally important in modulating iNKT cell function in immunometabolic disease.

## Author Contributions

FV, MB, and HS wrote the manuscript. FV and HS created the figures. BL and EK provided critical evaluation and offered insightful suggestions to improve the content. All edited the manuscript and approved the final version for submission.

## Conflict of Interest Statement

None of the authors have a competing financial interest in relation to the presented work. BL is employed by Nutricia Research and is leading a strategic alliance between University Medical Centre Utrecht/Wilhelmina Children’s Hospital and Nutricia Research, as indicated by the affiliations.
